# A rare case of fetus in fetu in the sacrococcygeal region: CT and MRI findings

**DOI:** 10.1186/s12887-021-03063-7

**Published:** 2021-12-15

**Authors:** Tao Lu, Junmei Ma, Xudan Yang

**Affiliations:** 1 Department of Radiology, Sichuan Provincial People’s Hospital, University of Electronic Science and Technology of China, 32 West Second Section, First Ring Road, Chengdu, 610072 Sichuan China; 2 Department of Pediatric surgery, Sichuan Provincial People’s Hospital, University of Electronic Science and Technology of China, 32 West Second Section, First Ring Road, Chengdu, 610072 Sichuan China; 3 Department of Pathology, Sichuan Provincial People’s Hospital, University of Electronic Science and Technology of China, 32 West Second Section, First Ring Road, Chengdu, 610072 Sichuan China

**Keywords:** Fetus in fetu, CT, MRI, Case report

## Abstract

**Background:**

Fetus in fetu is a rare condition in which a malformed fetus is found in the body of a living twin. The retroperitoneum is the most common location of this condition. However, the sacrococcygeal region is a rare site of the disease. The presence of vertebral bodies and limbs differentiates FIF from teratoma. Imaging modalities are important for diagnosing FIF.

**Case presentation:**

A 12-months old boy was hospitalized because of a mass in the sacrococcygeal region. CT showed a large, complex mass with bony structure resembling sacrococcygeal bone, hip bone and the femur in the sacrococcygeal region of the boy. The blood supply of the mass was from the aorta of the host. MRI revealed the mass was connected with the dilated sacral canal of the host, which resulted in tethered cord. A preoperative diagnosis of FIF was made and surgery was performed to remove the mass. Surgical removal and subsequent pathological examination revealed the anencephalic fetus had limb buds and a sacrum but no axial skeleton, which supported the diagnosis of FIF. Conclusions CT and MRI played important roles in diagnosing FIF based on the location of the lesion.

## Background

Fetus in fetu is a rare condition that can occur from the cranial cavity to the scrotal sac with the sacrococcygeal region the least common site of origin [1–3]. FIF is a congenital anomaly that is secondary to abnormal embryogenesis in a diamniotic, monochorionic and monozygotic twin [4]. Although preoperative diagnosis is based on radiologic findings, a definitive diagnosis is confirmed after surgery. Whether FIF is a separate disease or it is a form of highly differentiated teratoma is still controversial [5]. To the best of our knowledge, only 1 case of FIF in the sacrococcygeal region has been reported [6]. We report another rare case of FIF that occurred in the sacrococcygeal region and CT and MRI played an important role in diagnosing and evaluating of the disease.

## Case presentation

**Fig. 1:**
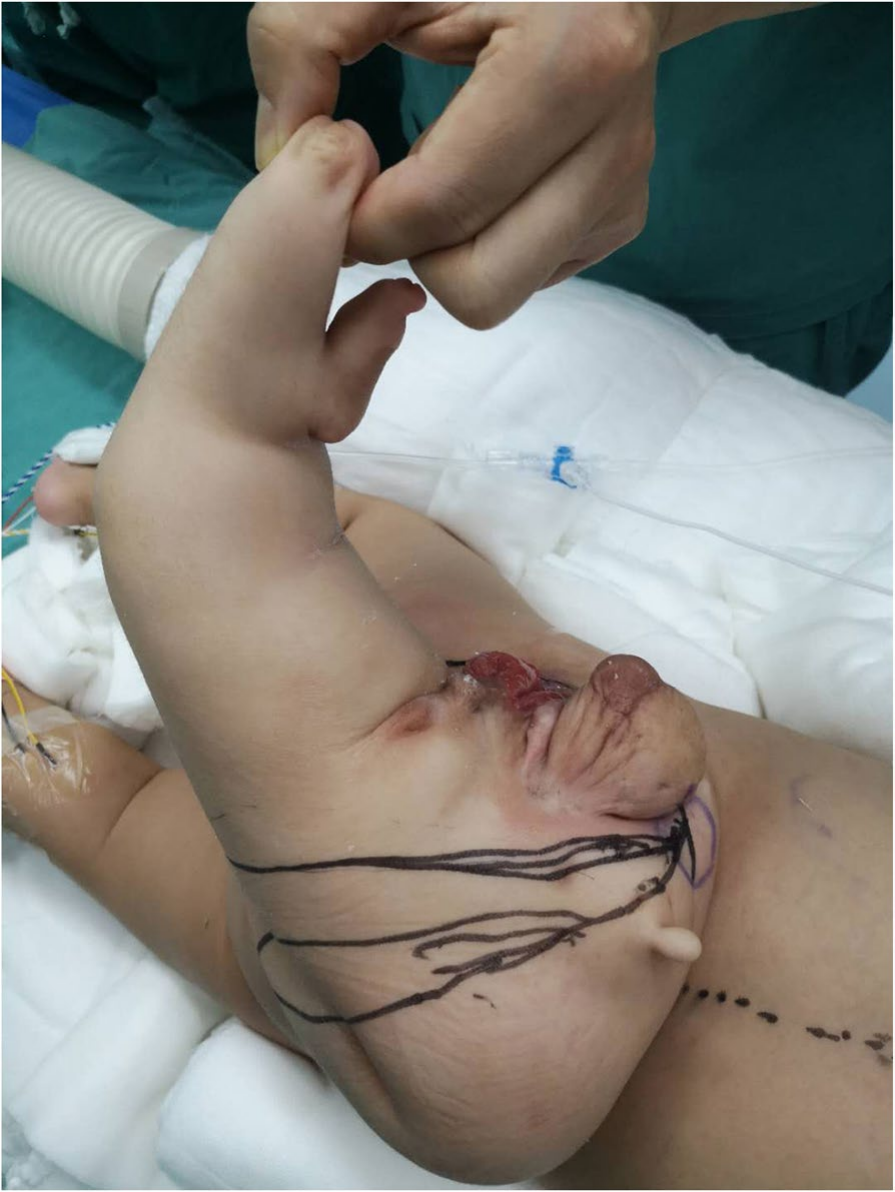
Clinical photogram demonstrated an irregular mass similar to a lower limb-like structure in the back and buttock of the boy

**Fig. 2:**
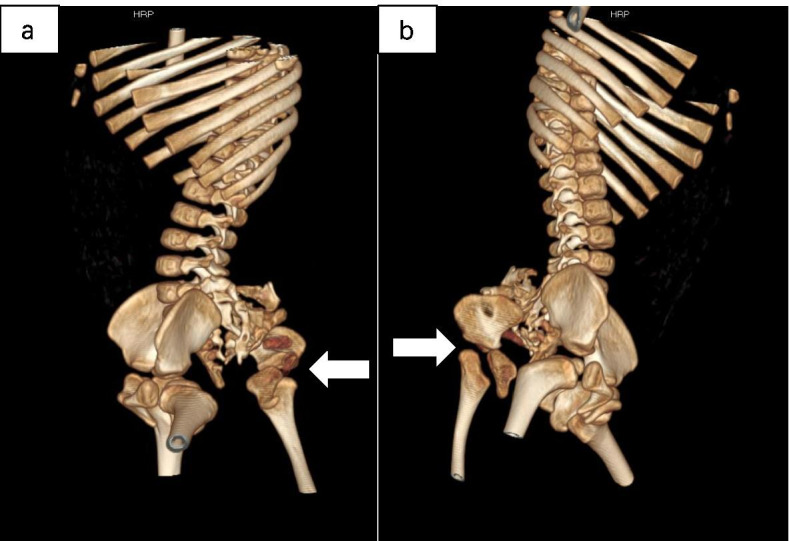
**a** and **b** 3D CT images showed the bony structure in the mass resembling sacrococcygeal bone, hip bone and the femur (thick arrow)

**Fig. 3:**
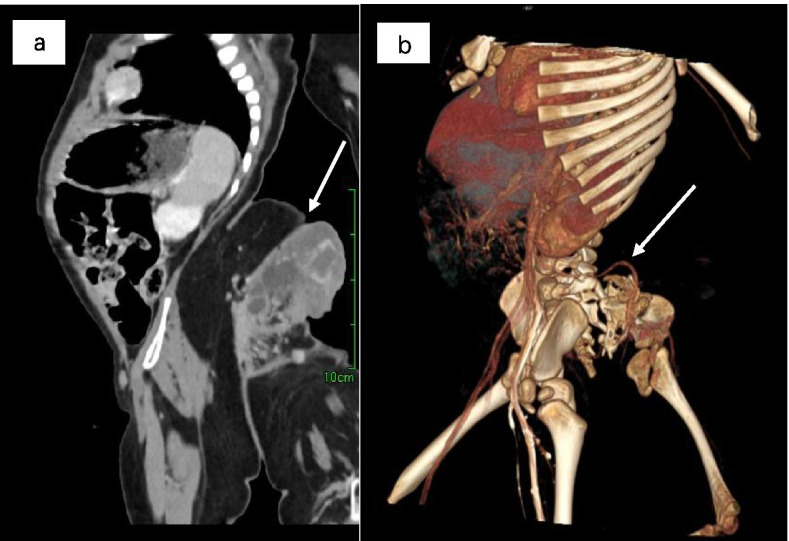
**a** and **b** sagittal and 3D CT images showed a large, complex mass with bony structure and blood supply from the aorta of the boy (arrow)

**Fig. 4:**
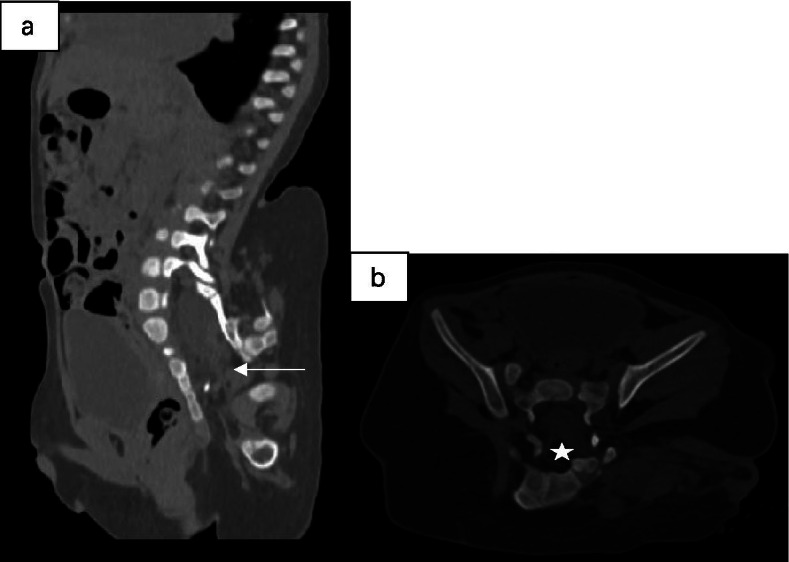
**a** and **b** sagittal and axial CT images showed Spinal dysraphisms of the sacrum (arrow and star)

**Fig. 5:**
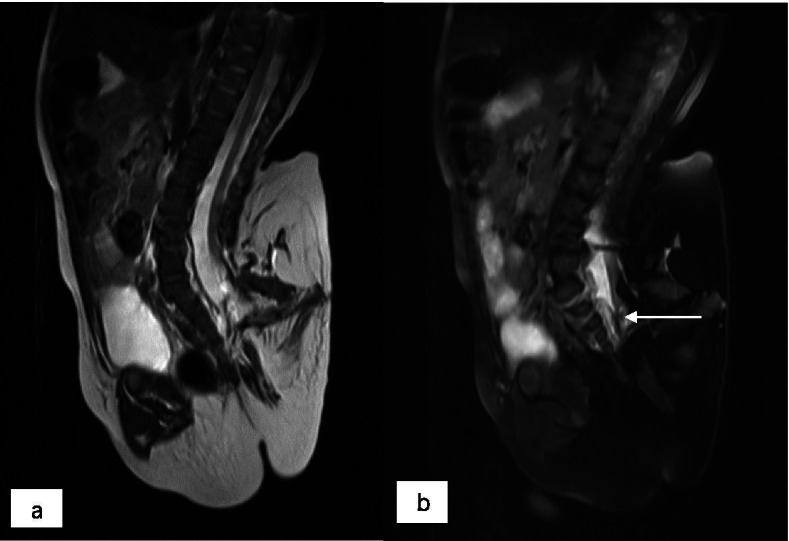
**a** and **b** sagittal T2WI (with and without fat suppression) showed the mass was connected with the dilated sacral canal of the boy and resulted in tethered cord. Note the thickened filum terminale was dragged posterior in the sacral canal (arrow)

A 12-months old boy was hospitalized because of a mass in the sacrococcygeal region. The boy was born full term by normal vaginal delivery with a birth weight of 3050 g. The sacrococcygeal mass was detected by prenatal US at 34 week’s gestational age. There was no family history of twins. The patient had no other congenital anomalies. On a physical examination, the boy was well-nourished, except for a lower limb-like structure that measured 35 × 25 cm, with a scrotum like structure in the lower back and buttock of the boy (Fig. 1). A complete blood count, kidney-liver function test, and tumor markers including β-HCG, AFP and CEA were within reference range. CT showed a large, complex mass with a bony structure resembling sacrococcygeal bone, hip bone and the femur (Fig. 2). The mass had cystic and solid components with blood supply from the aorta of the boy (Fig. 3). Spinal dysraphisms of the sacrum of the host were observed and adipose tissue in the epidural space was continuous with mass (Fig. 4). MRI showed the mass was connected to the dilated sacral canal of the boy. This resulted in a tethered cord and the conus medullaris was terminated at the level of the fourth lumbar vertebra. The filum terminale was thickened and had been moved to the sacral canal (Fig. 5). MRI also showed the mass had bony structure, cystic lesions, soft tissue components similar to skeletal muscle around joints and bones and abundant percutaneous adipose tissue. No anomalies in other systems including the urinary system, the cardiac system and the bowels were detected from abdominal CT, ultrasound, echocardiography and a Barium enema examination. No obvious mass effect was found because the mass was almost exophytic. A preoperative diagnosis of FIF was made and surgery was performed to remove the mass. Lysis of the tethered cord and sacral canal plasty were also performed. The patient’s parents agreed to perform the treatment that the boy received. On gross examination, the mass had malformed trunk, buttock, intestine and 1 lower limb with 1 toe in the foot. Upon incision, bones, soft tissues, malformed intestinal tissues were observed. Histopathological examination of the mass showed skin, nerves, bone and bone marrow, adipose tissue, skeletal muscle and intestinal tissue. The postoperative period was uneventful. The child was in good health during a follow-up of half a year and there was no evidence of recurrence.

## Discussion and conclusions

Willis described FIF as a rare entity in which a malformed parasitic twin resides in the body of its host, usually in the abdominal cavity [5]. The incidence of this rare condition is about 1 in 500,000 deliveries [7]. A total of 80% of FIF cases are located in the retroperitoneal space, while other cases are located in rare sites including the oral cavity, the cerebral ventricles, mediastinum, pelvis and the scrotum [1, 8–11]. FIF of our case is in the sacrococcygeal region, which is a relatively rare site of origin of the disease. However, the diagnosis of this case was easy because of the characteristic appearance of the exophytic, irregular mass.

### Presentations

FIF is usually detected in infancy and most of these cases are discovered before age of 18 months [12]. Asymptomatic cases with late presentations have also been reported, and the oldest reported patient with FIF was a 47-year-old man [13]. FIF also had a 2:1 male predominance, which is different from the female predominance of teratoma [14].

FIF is mostly anencephalic and the lower limbs are more developed than upper limbs in this condition. In almost all cases, the vertebral column and limb buds are present (91 and 82.5% respectively) [15]. In our case, CT showed that the mass contained an irregular bony structure similar to sacrococcygeal bone, hip bone and the femur, and these findings are consistent with previous reports.

Symptoms of FIF mainly result from its mass effect, including abdominal distension, dysphagia, emesis, feeding difficulties and jaundice [12] because most of cases are located in the abdomen. Dyspnea due to compression of lung by the mass and neurological symptoms associated with intracranial FIF have also been reported [16]. Our case was asymptomatic because the mass was in the sacrococcygeal region and mostly exophytic.

β-HCG and AFP levels are commonly used to rule out the development of germ cell tumors, especially the malignant teratoma. In our case, β-HCG and AFP levels were measured because the mass was in the sacrococcygeal region where a teratoma usually occurs. Although there is no evidence of a correlation between FIF and CEA, one case of FIF with elevated CEA level has been reported [16], which raised the concern for specific types of abdominal tumors. Whether this elevation was a manifestation of the FIF or an incidental finding is unclear. CEA levels were also measured in our case and the above-mentioned tumor markers were within reference range.

### Etiology

Two proposed theories mainly account for the etiology of FIF. One theory is the teratoma theory, which hypothesizes that FIF is a well-differentiated and highly organized mature teratoma [17]. FIF and teratoma may share an underlying pathogenetic mechanism because FIF usually occurs in the same sites as a teratoma, such as the retroperitoneum and ovaries [4]. FIF can also be associated with a teratoma. Retroperitoneal teratoma formation after resection of FIF and invertebrate teratomas containing well-developed fetiform structures have been reported [18, 19]. There are similarities at the histological level of these 2 entities. The other theory is the parasitic twin theory that proposes FIF is a monochorionic, monozygotic twin growing within its host twin [2, 12]. FIF is characterized by the presence of a complete or partial vertebral column and other appropriate situated axial or appendicular bones or organs [12]. The presence of vertebral bones suggests that FIF likely arises from a zygote at a primitive streak stage starting at the 3rd week in a normal embryo and in conjunction with gastrulation. These vertebral bones developed into the notochord which is the precursor of the vertebral column. Previous studies using DNA fingerprinting, molecular genetic analysis, and genotype and methylation analyses suggested that FIF is of monozygotic origin [20–22]. Miura’s study also showed different methylation patterns between a host infant and fetiform mass. However, the detailed methylation status at this locus during early human development is still unclear, so future studies on pathogenesis of FIF are required [20].

### Diagnosis

Imaging plays an important role in the preoperative diagnosis and follow-up of FIF. Ultrasonography and x-ray imaging are helpful in the detecting bony structure. US remains the first line imaging modality in prenatal and postnatal diagnosis because it is free of ionizing radiation and convenient. US was first used by Nicolini et al. to make a prenatal diagnosis of FIF and may be useful for diagnosing FIF as 21 weeks of gestation [15, 23]. CT with its 3D reconstruction technique can better display vertebral column and limb buds with long bones, and different components including soft tissue, fat and cysts, in the mass. Contrast-enhanced CT can further show feeding arteries and draining veins of FIF, and define the relationship between FIF and the adjacent organs and structures [24]. MRI is a versatile tool in depicting the complex components of FIF with its good soft tissue resolution and ability of multiplanar imaging.

The bony structure in the mass of our case supported the diagnosis of FIF instead of a teratoma. Although a teratoma consists of 3 primitive blastoderm cell layers, it does not develop through the primitive streak stage and does not have organgenesis or vertebral segmentation [25]. CT also detected spinal dysraphisms of the sacrum in the host and accumulation of adipose tissue in the epidural space. For the fetiform mass, CT not only detected bony structure of the mass, but also showed that the blood supply was from the infrarenal aorta of the host. MRI was adopted to demonstrate the relationship between the mass and the sacral canal of the host because the mass was located in the sacrococcygeal region. MRI was capable of showing a tethered cord in the host and multiple components of the fetiform mass. CT and MRI are required in the follow-up of our case as they have different advantages in showing different structures.

Because FIF grows in parallel with its host, surgical excision remains the treatment of the disease. The prognosis of FIF is favorable. However, clinical, radiological and serological follow-up is also recommended to detect recurrence owing to the presence of immature elements or malignancy because 1 case of malignant FIF has been reported [17].

## Conclusion

FIF is a rare congenital anomaly typically occurring in infants and children. When FIF manifested as an exophytic mass in the sacrococcygeal region, the patient may be asymptomatic. CT and MRI findings are helpful in the diagnosing this disease and in comprehensive demonstration of the anatomy of the mass and its surrounding structure. In particular, MRI should be used to demonstrate any anomalies with the sacral canal and conus medullaris of the host.

## Data Availability

The datasets used and/or analysed during the current study are available from the corresponding author on reasonable request.

## Abbreviations

FIF: Fetus in fetu
US: Ultrasonography
CT: Computer tomography
MRI: Magnetic resonance imaging
